# Impact of pseudouridylation, substrate fold, and degradosome organization on the endonuclease activity of RNase E

**DOI:** 10.1261/rna.078840.121

**Published:** 2021-11

**Authors:** Md. Saiful Islam, Katarzyna J. Bandyra, Yanjie Chao, Jörg Vogel, Ben F. Luisi

**Affiliations:** 1 Department of Biochemistry, University of Cambridge, Cambridge CB2 1GA, United Kingdom; 2 RNA Biology Group, Institute of Molecular Infection Biology, University of Würzburg, D-97080 Würzburg, Germany; 3 The Center for Microbes, Development and Health (CMDH), Institut Pasteur of Shanghai, Chinese Academy of Sciences, Xuhui district, Shanghai, 200031, China; 4 Helmholtz Institute for RNA-based Infection Research (HIRI), Helmholtz Centre for Infection Research (HZI), D-97080 Würzburg, Germany

**Keywords:** modified RNA, riboregulation, RNA recognition, ribonuclease mechanism, pseudouridine, RNA degradosome

## Abstract

The conserved endoribonuclease RNase E dominates the dynamic landscape of RNA metabolism and underpins control mediated by small regulatory RNAs in diverse bacterial species. We explored the enzyme's hydrolytic mechanism, allosteric activation, and interplay with partner proteins in the multicomponent RNA degradosome assembly of *Escherichia coli.* RNase E cleaves single-stranded RNA with preference to attack the phosphate located at the 5′ nucleotide preceding uracil, and we corroborate key interactions that select that base. Unexpectedly, RNase E activity is impeded strongly when the recognized uracil is isomerized to 5-ribosyluracil (pseudouridine), from which we infer the detailed geometry of the hydrolytic attack process. Kinetics analyses support models for recognition of secondary structure in substrates by RNase E and for allosteric autoregulation. The catalytic power of the enzyme is boosted when it is assembled into the multienzyme RNA degradosome, most likely as a consequence of substrate capture and presentation. Our results rationalize the origins of substrate preferences of RNase E and illuminate its catalytic mechanism, supporting the roles of allosteric domain closure and cooperation with other components of the RNA degradosome complex.

## INTRODUCTION

RNase E, a key bacterial endoribonuclease of ancient evolutionary origin, has multifaceted activities critical to organism fitness, including the turnover of mRNA, maturation of precursors of tRNA and rRNA, processing and degradation of small regulatory RNAs, and rRNA quality control (Mackie 1998, 2013; Bandyra et al. 2013). Once cleaved by RNase E, an mRNA becomes committed to an irreversible fate of rapid deconstruction; but at the same time, the enzyme can contribute to an orderly genesis of structured RNAs from precursors that circumvents destructive pathways, provided that those species satisfy quality control checks. The enzymatic activity of RNase E, which appears to be nuanced, serves as a key determinant of cellular RNA lifetime in cells. Its substrate preferences and encounter rate with RNA impact on transcript lifetime in vivo and are of interest for elaborating a potential code that could define cellular RNA fate.

Decades of analysis of RNase E activity indicate that there is no simple sequence code for its substrates per se, but instead a strong preference to cleave within single-stranded regions enriched in A or U (Kime et al. 2010, 2014; Mackie 2013; Del Campo et al. 2015; Chao et al. 2017). Global RNA target analyses performed both in vivo and in vitro identify uracil positioned to the 3′ side adjacent to the nucleotide of the scissile phosphate (the +2 position) as a strong signature for RNase E activity (Chao et al. 2017). For many substrates that follow either destructive or maturation pathways, the enzyme is activated by transformation of the 5′ end of the substrate from a triphosphate normally found on nascent transcripts, to a monophosphate found on processed species (Mackie 2013). For other substrates, the status of the 5′ end is not so critical for RNase E action (Baker and Mackie 2003; Clarke et al. 2014; Kime et al. 2014), and for these “5′ end bypass” substrates, other features such as secondary structure of the RNA appear to be important. Secondary structure contributes to recognition of sites for cleavage in both degradative and processing pathways (Bandyra et al. 2018; Updegrove et al. 2019; Richards and Belasco 2021).

**FIGURE 1.**
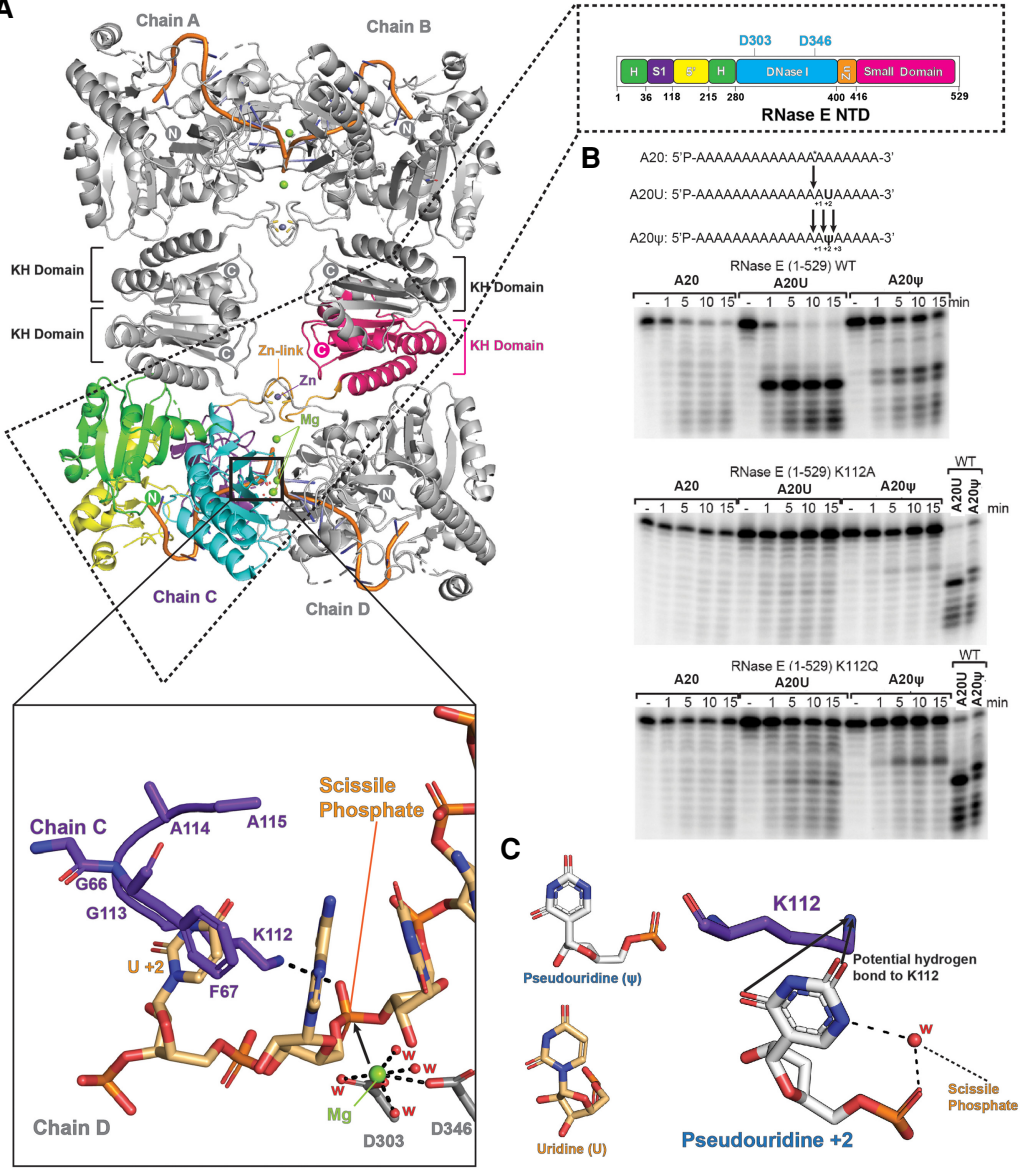
Role of RNase E K112 in interaction with uracil +2 of the substrate, and impact of pseudouridylation. (*A*) The tetrameric RNase E catalytic domain (NTD) in complex with RNA (PDB: 2C0B) (Callaghan et al. 2005). The inset on the *upper right* shows a cartoon schematic of the domains showing active-site residues D303 and D346. The *lower inset* shows a model of the organization of binding of RNA substrate based on the structure of chemically protected RNA (PDB 2C0B). The residues in purple are from the S1 domain of RNase E and the scissile phosphate from the RNA bound in the active site on the interface of two protomers presented for the hydrolytic attack by the waters associated (W, red) with magnesium ion (Mg^++^, green sphere); the U+2 is proposed to be sandwiched between side chains of amino acids K112 and F67. (*B*) Cleavage assays of RNase E. Cleavage of 20-mer polyadenine (A20), polyadenine with an uracil at position 15 (A20U), and polyadenine with a pseudouridine (ψ) at position 15 (A20ψ) by wild-type RNase E NTD (*top* panel), RNase E NTD with a mutation of K112A (*middle* panel), and K112Q (*bottom* panel). The substrate was 5′ end-labeled and the products were resolved on denaturing urea-PAGE gels. The time points of the reactions are annotated *above* the gels. (*C*) A proposed model of the likely hydration organization at the site of pseudouridine (ψ). The model also shows a probable hydrogen-bond mediated interaction between K112 and pseudouridine. A crystal structure of a duplex RNA (PDB 3CGS) was used to make the model.

The critical endonuclease activity of RNase E is encompassed within the highly conserved amino-terminal domain (NTD) (Fig. 1A), which corresponds to roughly half the protein mass. Crystallographic studies of this domain have provided insight into the origins of substrate recognition and 5′-end dependent activation (Fig. 1A; Callaghan et al. 2005; Koslover et al. 2008; Bandyra et al. 2018). Key structural motifs of the NTD include an RNA binding S1 domain and a 5′-sensor that can read the chemical status of the RNA 5′-end (Fig. 1A). The recognition of the 5′-end triggers a conformational switch that maneuvers the S1 domain to clamp onto substrates and present them in the active site with geometry favorable for hydrolytic attack. A zinc-coordination motif links two protomers into a dimer, and two such dimers self-associate through a small domain that is evolutionarily related to the KH RNA binding module (Fig. 1A; Pereira and Lupas 2018). A vestigial RNase H-like subdomain has no catalytic activity but has been observed to cooperate with the KH-like small domain to recognize duplex structures in substrates and help present adjacent single-stranded regions to the proximal active site (Fig. 1A; Bandyra et al. 2018). Surprisingly, the enzyme is driven into a hyperactive state by simple substitutions in a conserved pocket of this domain that correspond to nearly single-atom replacement (D26N, D28N, D338N; hereafter NTD-3M) (Bandyra et al. 2018; Updegrove et al. 2019). These observations support a model in which the RNase H-like domain auto-regulates the activity of the enzyme by influencing the energetics of domain closure (Bandyra et al. 2018).

The carboxy-terminal half of the protein, which is predicted to be intrinsically disordered (Aït-Bara and Carpousis 2015; Aït-Bara et al. 2015; Callaghan et al. 2004), provides the scaffold to assemble protein partners into the RNA degradosome complex (Bandyra et al. 2013, 2018; Bruce et al. 2018). Through the cooperation of its components and recruitment of RNA chaperones such as Hfq, the RNA degradosome is the central machinery in *Escherichia coli* and many other species for processing of structured precursors and turnover of RNA. RNase E also contains a short amphipathic α-helical domain that interacts with the *E. coli* inner membrane, and the resulting membrane localization of the degradosome adds a spatial layer to post-transcriptional gene regulation (Khemici et al. 2008; Mackie 2013; Hadjeras et al. 2019). Two RNA binding sites in the carboxy-terminal domain of RNase E, referred to as AR1 and AR2, cooperate with the RNA helicase RhlB to assist in substrate unwinding and remodeling (Leroy et al. 2002; Khemici and Carpousis 2004; Chandran et al. 2007; Garrey et al. 2009). The two RNA binding sites, together with RhlB can interact with ribosomes (Tsai et al. 2012) and may enable the degradosome to cleave mRNA in support of a proposed scavenging process (Deana and Belasco 2005; Dreyfus 2009). A plausible scenario is that the close proximity of the RNA degradosome to the translational machine prevents the translation of aberrant transcripts and rescues stalled ribosomal assemblies as part of bacterial RNA surveillance.

Open questions remain regarding details of the RNase E catalytic mechanism, and its capacity to act on modified RNA. The effect of the interplay between the components of the degradosome on the quantitative activity of the catalytic domain also have not been evaluated. In this report, we measured the ribonuclease activity of RNase E and its variants that affect substrate recognition, and we explored how the RNA degradosome assembly cooperates with this activity. Analysis of RNase E activity on substrates with pseudouridine shows that, surprisingly, the enzyme is very sensitive to this modification. Taken together, our results provide mechanistic insights into RNase E catalytic mechanism, allostery, and cooperation within the RNA degradosome complex.

## RESULTS

### K112 plays an important role in substrate preference and cleavage by RNase E

Modeling using the crystal structures of the amino-terminal catalytic domain (NTD) of RNase E predicted that S1 domain residues K112 and F67 interact with the base at position +2 to orient the single stranded region of the RNA substrate into a favorable geometry at the active site for nucleophilic attack by water (Fig. 1A; Chao et al. 2017). Uracil at the +2 position is predicted to be favored by a hydrogen bonding interaction between the amino group of K112 and the exocyclic carbonyl groups that contributes to the sequence preference at that position. Based on the X-ray structure of RNase E with modified RNA (Callaghan et al. 2005), the +2 base is also predicted to be sandwiched between the aromatic ring of F67 and the aliphatic component of the K112 side chain (Fig. 1A). The orientation for the K112 side chain to make the base-sandwiching interaction may differ from that to make the hydrogen bond to U+2, and it may switch conformation during the catalytic process so that its amino group may interact with the phosphate to stabilize the charge of the hydrolytic intermediate.

To test the importance of K112, we compared activities of purified wild-type and mutant versions of NTD using a model single-stranded RNA substrate composed of 20 adenine residues (A20) and its uracil variant with a single uracil at position 15 (A20U) (Fig. 1B). The time course for the cleavage is shown in Figure 1B, with products resolved on an RNA denaturing gel. At the enzyme:substrate ratios used in these assay conditions, corresponding to multiple turnover conditions, RNase E NTD cleaves efficiently at the phosphate 2 nt upstream of uridine, consistent with the U+2 ruler-and-cut mechanism (Chao et al. 2017). The cleavage rate of the uracil-containing substrate is higher compared to the substrate with no uracil (Fig. 1B, top panel, compare A20 and A20U). When K112 is substituted with alanine, the enzyme activity and specificity are greatly diminished for the uracil-containing substrate, with more starting substrate remaining over the time course and the degradation pattern resembling a uniform ladder, as distinct from being enriched for a particular species (Fig. 1B, middle panel, compare A20 and A20U with top panel). Even the comparatively conservative substitution of K112 with the long polar side chain of glutamine has diminished cleavage preference for the U+2 position (Fig. 1B, bottom panel). In general, substitution of lysine by the polar glutamine is expected to retain capacity for hydrogen bond formation. However, based on the crystal structure, the glutamine is predicted to be too short to hydrogen bond with either the uracil carbonyl groups or the phosphate backbone. These results corroborate the importance of the K112 interaction for catalysis and suggest that the hydrogen bonding interaction with either the uracil base or the scissile phosphate or both are required for optimal activity.

### Pseudouridine impedes RNase E activity and shifts the cleavage site

The substitution of the uracil at position +2 with pseudouridine (ψ) involves an isomeric transformation of the base and was not expected to impact the presentation of the hydrogen bonding groups of O2 and O4 (Fig. 1C). However, pseudouridine showed a profound effect on the cleavage activity of RNase E (Fig. 1B, top panel, compare A20U with A20ψ). Most of the pseudouridine containing substrate resisted cleavage by RNase E in the course of the experiment. The cleavage site seems to be shifted relative to the cleavage when uridine is present. These findings suggest that the recognition of uracil is not simply due to a hydrogen bonding interaction with the principal substituents of the base, but also depends on the detailed interactions that influence the phospho-diester geometry (Westhof 2019). The substitution of U with pseudouridine may affect the hydration pattern of the substrate and the energy required to achieve the conformation that enables development of the enzymatic transition state (Fig. 1C; Charette and Gray 2000).

The substitution of K112 with Q, which impedes activity of the wild-type enzyme, changed the cutting pattern of the pseudouridine containing substrate. The preferred cleavage site of the K112Q mutant protein moved to the position +2 relative to the cut-site for the wild-type counterpart (Fig. 1B, top and bottom panels). A lesser degree of cleavage of pseudouridine containing substrate was also observed for the K112Q mutant. The overall reduction in cleavage rate along with a shift in preferred cleavage site suggests that the activation of hydrolysis requires a long positively charged or polar side chain at position 112 (Fig. 1A). The K112Q substitution perhaps causes the substrate to align differently in the active site pocket so that it is shifted by one or two nucleotides in the 3′ direction compared to the corresponding wild-type complex.

### RNase E catalytic power can be boosted by substitutions at DNase I and RNase H-like domains

**FIGURE 2.**
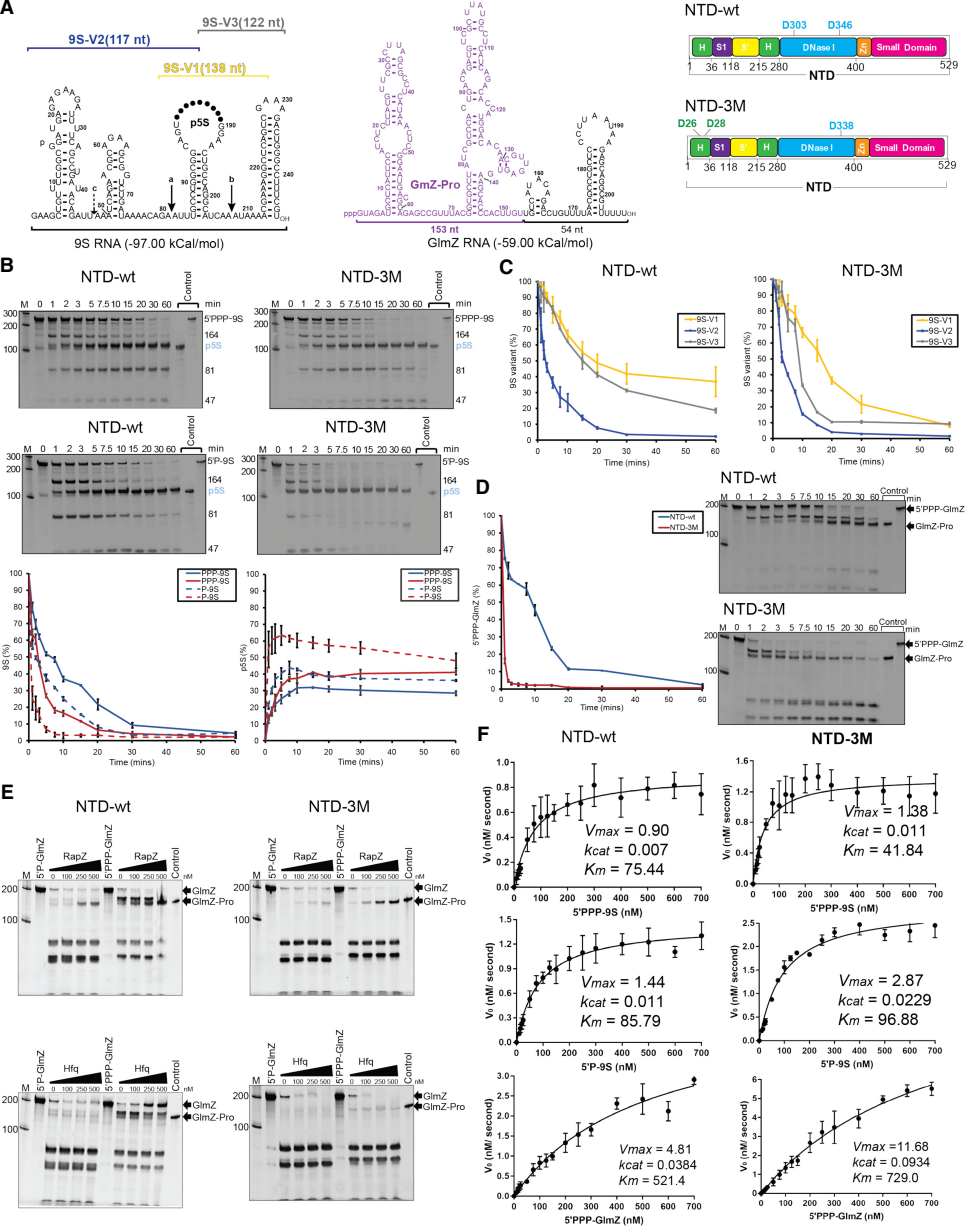
Mutations in the RNase H-like and DNase I domains improve catalytic efficiency of RNase E. (*A*) The *left* shows a schematic of secondary structure of 9S RNA with three cleavage sites marked as “*a,*” “*b,*” and “*c*” (Christiansen 1988; Lorenz et al. 2011); the bars *above* the schematic show the three segments (9S-V1, 9S-V2, and 9S-V3) generated for cleavage assays. The *middle* panel shows secondary structure of GlmZ RNA predicted by the ViennaRNA Package 2.0 (Lorenz et al. 2011). The *right* panel shows an annotated domain schematic for NTD-wt and NTD-3M harboring mutations in RNase H-like (D26N and D28N) and DNase I (D338N) domains. (*B*) Denaturing RNA gels showing time course cleavage assay of 9S (5′-triphosphorylated, *upper* panel; 5′-monophosphorylated, *lower* panel) using NTD-wt (blue lines) and the NTD-3M (red lines). The *lower* panel shows the integrated signal for 9S (*left*) and p5S product (*right*). (*C*) Integrated signal for the 9S segments V1, V2, and V3 obtained against NTD-wt and NTD-3M. (*D*) Integrated signal for GlmZ cleavage over time for NTD-wt and NTD-3M shown on the *left* panel with the corresponding denaturing gels shown on the *right*. (*E*) Denaturing RNA gels for GlmZ processing by NTD-wt and NTD-3M in the presence of RapZ or Hfq, showing the production of GlmZ-Pro is sensitive to the presence of RapZ but not Hfq. (*F*) Michaelis–Menten plots used to determine the kinetics parameters of cleavage of 9S and GlmZ RNAs. The plots were fitted using Prism (GraphPad Software) and represent mean of three representative plots of reaction rates versus substrate concentrations (see “Materials and Methods” for details). (H) RNase H-like domain, (S1) RNA binding S1 domain, (DNase I) DNase I-like domain, (5′) RNA 5′ site-sensing pocket, (Zn) Zn-linker.

Earlier studies showed that the catalytic activity of RNase E is boosted by mutations of conserved, non-catalytic residues in the RNase H-like domain (D26N and D28N) and DNase I domain (D338N) (Fig. 2A, right panel; Bandyra et al. 2018). The substitutions are at a distance from the active site but involve regions where the conformational changes associated with apo to substrate-bound states occur and are likely to impact on the allosteric switching of the enzyme (Bandyra et al. 2018). We compared the catalytic activity of the wild-type (NTD) and the hyperactive variant carrying mutations at positions D26, D28, and D338 where all three aspartate residues were mutated to asparagine (NTD-3M). For substrates, we used GlmZ, which is a regulatory sRNA that gets inactivated by RNase E cleavage, and 9S RNA which is a precursor of ribosomal 5S RNA (Fig. 2A).

The enzyme cleaves the 9S mainly at three sites to form the p5S precursor ribosomal RNA product (Fig. 2A; Christiansen 1988; Cormack and Mackie 1992; Carpousis et al. 1994). Stem–loop II has previously been shown as the minimal structural requirement needed for RNase E to cleave at the “*a*” site (Fig. 2A; Cormack and Mackie 1992; Carpousis et al. 1994; Mackie 2013). We also generated three segments of 9S RNA encompassing different predicted secondary structures (indicated by bars above the 9S schematic in Fig. 2A). Version 1 has cut-sites “*a*” and “*b*” and is similar to the 9Sa substrate previously investigated by others (Carpousis et al. 1994). Version 2 has cut-sites “*a*” and “*c*,” and version 3 encompasses only cut-site “*b*” (Christiansen 1988). The cleavage assays with 9S and its truncated versions confirm earlier observations (Mackie and Genereaux 1993; Carpousis et al. 1994) that RNase E action can be influenced by the secondary structures upstream and downstream to the recognition site (Fig. 2C, left panel).

Compared to the wild-type enzyme, NTD-3M showed higher activity for all substrates tested. Its activity is shown for the 9S substrate in Figure 2B, for the three smaller constructs of 9S in Figure 2C, and for the GlmZ sRNA in Figure 2D. These results suggest that the activity enhancement of NTD-3M does not require a specific sequence or RNA fold. GlmZ cleavage by RNase E is guided by the protein RapZ, which has high specificity for the guiding effect and is not observed with the RNA chaperone Hfq (Fig 2E; Supplemental Fig. S1; Kalamorz et al. 2007; Urban and Vogel 2008; Göpel et al. 2013; Gonzalez et al. 2017; Durica-Mitic and Görke 2019). In the presence of NTD-3M, the guiding effect of RapZ is enhanced, but the GlmZ cleavage is either inhibited or proceeds nonspecifically without forming the GlmZ-Pro product in the presence of Hfq (Fig. 2E).

The 5′ phosphorylation state of 9S RNA can impact on the first cleavage events, with the second event having the activating 5′P group present and anticipated to be intrinsically accelerated if the group is read by the enzyme (Cormack and Mackie 1992; Mackie 2013). For the 9S substrate, the status of the 5′ end affects the rate of disappearance of the 9S band and generation of the p5S product (graphs in lower panel, Fig. 2B), with a boost seen for 5′ monophosphate versus triphosphate, corroborating earlier findings that 5′-sensing can contribute to the first cleavage event in 9S processing by RNase E (Cormack and Mackie 1992; Mackie 2013). This boosting effect is also seen for the NTD-3M mutant and suggests that the mutation does not impact on 5′ sensing.

For all substrates tested, a boost in catalytic power was observed, due to both increased catalytic rate and decreased *K*_*m*_ (Table 1). Taken together, these results support the proposed role of allosteric autoregulation of enzyme activity (Bandyra et al. 2018), in which domain closure helps to preorganize the active site so that the apparent affinity of the Michaelis–Menten complex increases probably by decreasing the energy barrier to capture and engulf the substrate.

**TABLE 1.**
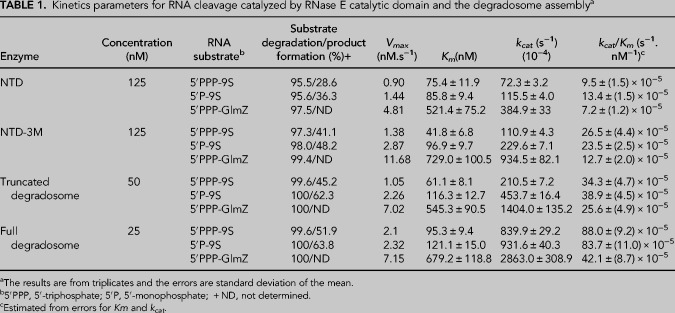
Kinetics parameters for RNA cleavage catalyzed by RNase E catalytic domain and the degradosome assembly^a^

### Metals in the catalytic mechanism: RNase E active site may recruit one metal in the apo form

**FIGURE 3.**
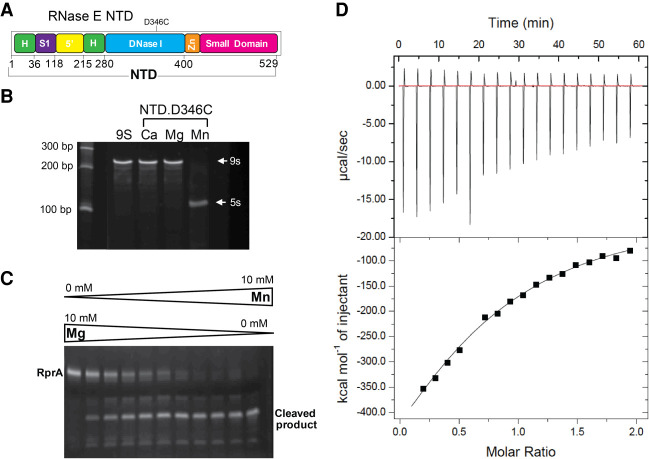
Metal interactions in the active site of RNase E. (*A*) Schematic of RNase E NTD showing mutant D346C in the active site. The mutant is catalytically active in the presence of Mn^++^ but not any other metal as seen for processing of 9S and sRNA RprA (*B*,*C*, respectively). (*D*) An isothermal calorimetry titration curve for NTD.D346C interactions with Mn^++^. The *K*_*D*_ is 17 µM for Mn^++^. The titration curve is representative of three independent experiments.

The active site bears two conserved aspartate residues (D303 and D346) that recruit magnesium ion to activate a water molecule for nucleophilic attack on the scissile phospho-diester bond (Fig. 1A; Callaghan et al. 2005; Thompson et al. 2015). One question relevant to the mechanism is whether metal is bound to the site in the apo form or if metal binding requires substrate. The binding interactions between RNase E and metal cofactor were evaluated by isothermal calorimetry (ITC) using a variant of RNase E with residue D346 replaced with a cysteine residue, which was reported previously to be catalytically active in the presence of Mn^++^, but not Mg^++^ (Fig. 3A; Thompson et al. 2015). Testing the activity of NTD.D346C on two different RNAs, 9S and the small RNA RprA, confirms that the enzyme is active for cleavage only in the presence of Mn^++^ (Fig. 3B,C). Using isothermal titration calorimetry (ITC) and titrating the mutant enzyme against Mn^++^ yields a *K*_*D*_ for metal binding in the absence of RNA at 17 µM, with associated ΔH = −19.45 kcal/mol and ΔS = −35.4 cal/mol/deg (Fig. 3D). The binding profile indicates that one metal ion can be bound by each subunit of the catalytic domain in the absence of substrate.

### Probing the RNase E mechanism with unnatural amino acids

**FIGURE 4.**
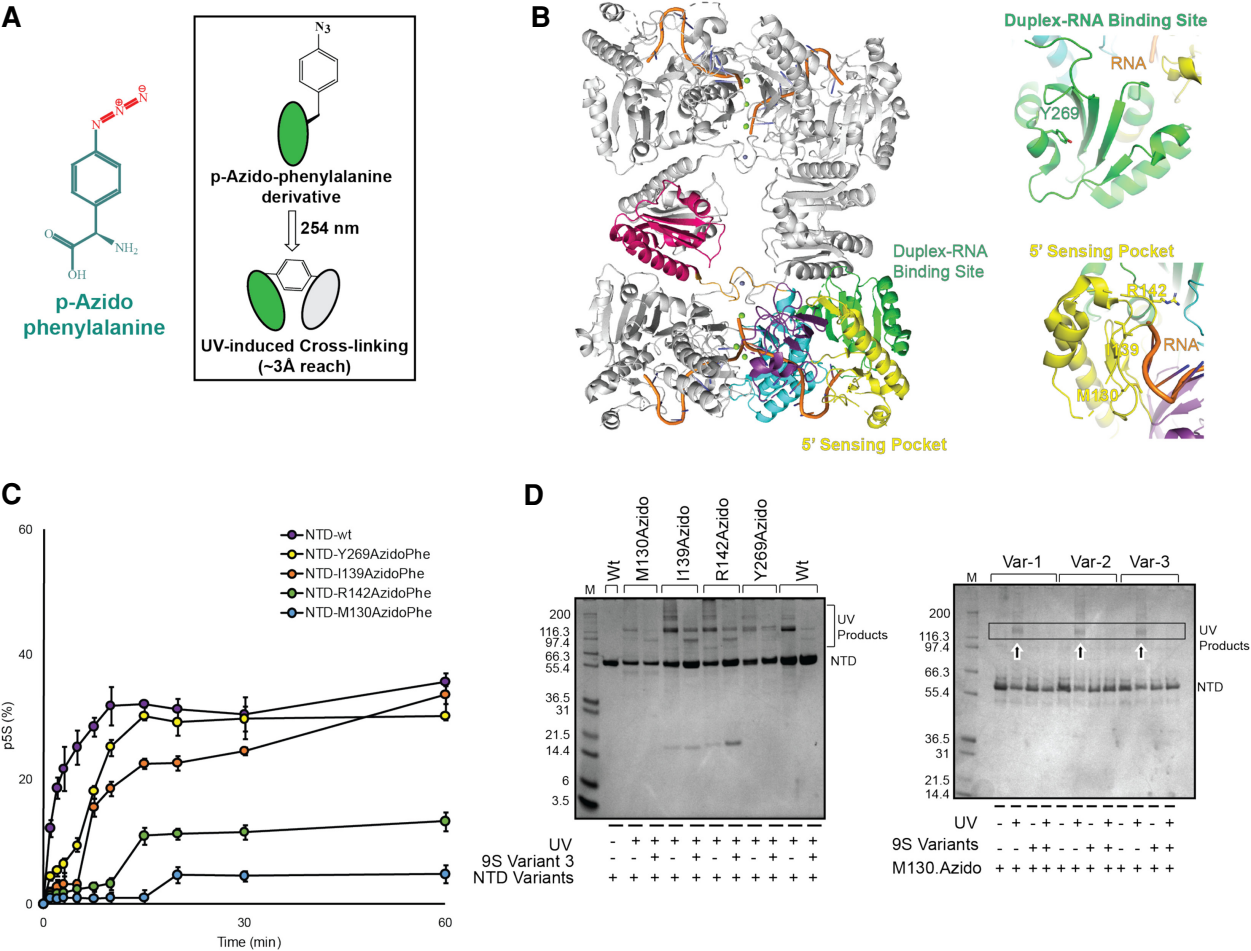
Incorporation of azido-phenylalanine into the RNase E catalytic domain. (*A*) Chemical formula of para-azido-phenylalanine (p-AzidoPhe); *inset* shows p-AzidoPhe photo-crosslinking to nearby residues upon exposure to UV light at 254 nm. (*B*) Models of RNase E NTD tetramer with bound RNA at active site, 5′ sensor and the duplex recognition region with *insets* showing the residues (M130, I139, R142, and Y269) substituted with p-AzidoPhe (model based on PDB 2C0B). (*C*) Time course assay of p5S production from 9S RNA, processed by p-AzidoPhe derivatives of NTD; values represent mean (*n* = 3) and standard deviation. (*D*) Denaturing protein gels showing p-AzidoPhe derivatives of RNase E NTD form UV cross-linked product(s). The p-AzidoPhe modified protein may form intradomain interaction(s) upon light exposure which are lost in the presence of 9S RNA, suggesting masking of the crosslinking moiety upon RNA binding.

To further explore interactions between the catalytic NTD and its substrates, we prepared derivatives of the protein with the photo-crosslinkable amino acid para-azido-phenylalanine (p-AzidoPhe) incorporated at specific positions in the 5′-sensing pocket and the duplex-RNA binding site using the amber suppressor system (Fig. 4A; Chatterjee et al. 2014). Single substitutions were made at residues M130, I139, R142 in the 5′-sensing pocket and Y269 on the duplex binding surface (Fig. 4B). Surprisingly, time course activity assays indicated that formation of the p5S species from 9S becomes impeded by all three substitutions in the 5′-sensing pocket, suggesting that the changes perturb RNA interactions (Fig. 4C). On the other hand, the Y269 substitution at the duplex binding surface showed little impact on activity (Fig. 4C). Exposing NTD p-AzidoPhe derivatives to light at 254 nm in the presence of the 9S segments indicated schematically in Figure 2A did not yield photo-crosslinking directly to the RNA that could be detected by mobility shifts in denaturing protein gels (Fig. 4D). However, the protein migrated differently in the denaturing gel upon UV light illumination in the absence of RNA, and this may arise from intramolecular crosslinks that either change or become masked upon RNA binding (Fig. 4D). While these results did not yield the RNA–protein adducts that were anticipated, they demonstrate the feasibility of introducing unnatural amino acids into RNase E for future studies and also highlight the sensitivity of the 5′-sensing pocket to mutations that impact on processing activity.

### Activities of the degradosome for cleavage of complex substrates

**FIGURE 5.**
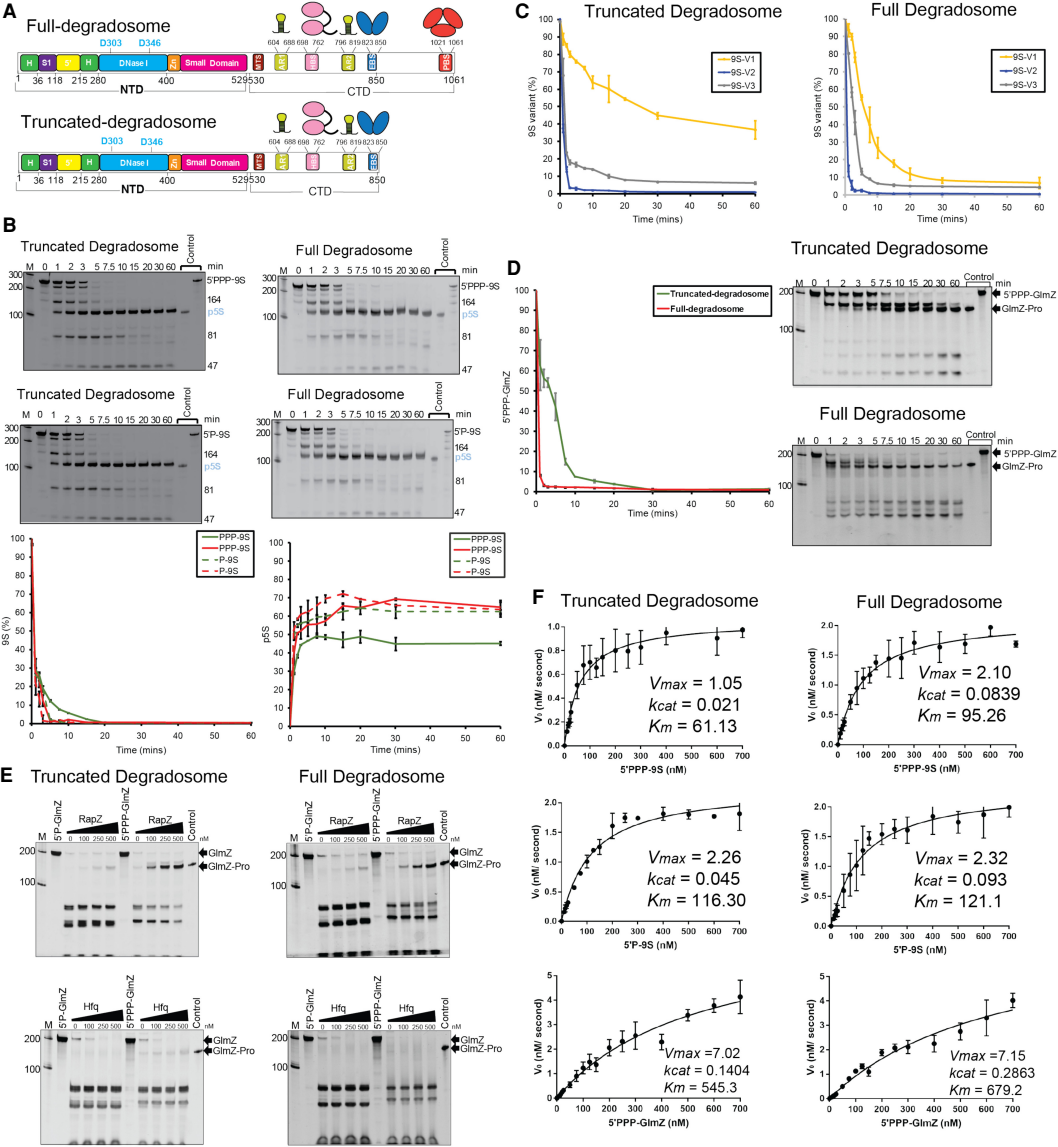
Substrate cleavage catalyzed by the RNA degradosome complex. (*A*) Schematics of the full degradosome (RNase E, enolase, RhlB, and PNPase) and truncated degradosome (RNase E, enolase, and RhlB) assemblies. (*B*) Time course cleavage assay showing processing of 9S RNA and production of the precursor RNA p5S for 9S RNA with 5′-triphosphate (PPP-9S, *upper* panel) and 5′-monophosphate (P-9S, *middle* panel). The *lower* panel shows integrated signal for 9S signal loss (plot on the *left*) and p5S signal gain (plot on the *right*) from 9S cleavage assays catalyzed by truncated degradosome (green lines) and full degradosome (red lines). (*C*) Plots of cleavage of 9S subdomains (9S-V1, 9S-V2, and 9S-V3) catalyzed by degradosome assemblies. (*D*) Plots of cleavage of GlmZ RNA catalyzed by the degradosome assemblies with the denaturing gels used to quantify signals shown on the *right*. (*E*) Denaturing gels showing the production of GlmZ-Pro by RNase E is sensitive to the presence of RapZ but not Hfq within the degradosome assembly too. (*F*) Michaelis–Menten plots used for determination of the kinetics parameters of the cleavage of 9S and GlmZ RNAs catalyzed by truncated degradosome and full degradosome. The plots were fitted using Prism (GraphPad Software) and represent mean of three representative plots of reaction rates vs substrate concentrations (see “Materials and Methods” for details). (H) RNase H-like domain, (S1) RNA binding S1 domain, (DNase I) DNase I-like domain, (5′) RNA 5′ site-sensing pocket, (Zn) Zn-linker, (MTS) membrane targeting site, (AR) Arginine-rich region/RNA binding site, (HBS) RhlB binding site, (EBS) Enolase binding site, and (PBS) PNPase binding site.

To explore how RNase E activity is impacted by the degradosome organization, we studied the activity of the assembly to cleave 9S and GlmZ. Purified recombinant degradosome (comprising RNase E 1-1061, RhlB, enolase, and PNPase) was prepared, as well as a subassembly comprising RNase E 1-850, RhlB, and enolase (truncated degradosome; Fig. 5A). The activity for processing of 9S was relatively greater for the truncated degradosome and full degradosome assemblies compared to the isolated catalytic domain under identical experimental conditions (Table 1; Fig. 5B). The cleavage rates were also seen to be greater for 5′P-9S compared to 5′PPP-9S (Fig. 5B). Increased activity was also observed for the 9S segments (Fig. 5C) and processing of GlmZ (Fig. 5D). As seen with the results with the NTD, RapZ has a guiding effect on cleaving GlmZ, but Hfq does not (Fig. 5E; Supplemental Fig. S1).

The degradosome shows increased catalytic power (*k_cat_/K*_m_) compared to the NTD for all substrates, mostly through changes to *k*_*cat*_ (Table 1, Fig. 5F). The degradosome assembly has several RNA binding sites that may help to capture and channel substrates (Dendooven et al. 2021), perhaps combined with better organization of the RNase E active site that potentiates domain closure and ensuing catalytic activity.

## DISCUSSION

In many bacterial species, the half-lives of most transcripts are defined by the activity of RNase E, and sequence and structural preferences for substrates have been identified from in vitro and in vivo experiments (Mackie 2013; Clarke et al. 2014; Kime et al. 2014; Chao et al. 2017). Here, we explored the activity of RNase E on different RNAs to gain further insight into substrate recognition and cooperation between domains and partner proteins. We quantified metal interaction and impact of allosteric mutations and degradosome assembly on activity. The impact of substrate modification by pseudouridylation had not been addressed in earlier studies and was studied here.

The cleavage assays with 9S and its truncated versions confirm that RNase E action can be influenced by secondary structures upstream and downstream from the cleavage site. Cleavage of all investigated RNAs is influenced by the RNA degradosome assembly. Corroborating earlier findings, mutation of conserved aspartates to asparagines in the RNase H-like subdomain boosts hydrolytic activity (Bandyra et al. 2018). A higher reaction rate for the NTD-3M mutant with lower *K*_*m*_ and higher *k*_*cat*_ suggest that the RNase H-like and DNase I domains help to cleave RNAs by increasing the catalytic power of the enzyme. As these domains switch conformation with substrate binding, it is possible that they can impact on product release, with the mutant acting more quickly than wild-type for this step.

The results presented here corroborate the importance of uracil at position +2 with respect to the cleavage site as a key feature of a preferred cleavage site by RNase E and the role of residue K112 in recognizing the +2 uracil. Unexpectedly, cleavage by RNase E is strongly impeded when the +2 uracil is substituted with pseudouridine, which is surprising given that this substitution presents only one new hydrogen bonding group on the pyrimidine. The isomerization of uracil to pseudouridine presents the N1 as a hydrogen bond donor and may affect the hydration pattern that will include interaction with the phosphate backbone. In most RNA structures, N1 is predicted to interact with the phosphate backbone of both the pseudouridine and the 5′ residue (Westhof 2019; Charette and Gray 2000). In the context of the RNase E catalytic site, this interaction could restrict the backbone conformation at position +2 and disfavor the geometry necessary for catalysis.

**FIGURE 6.**
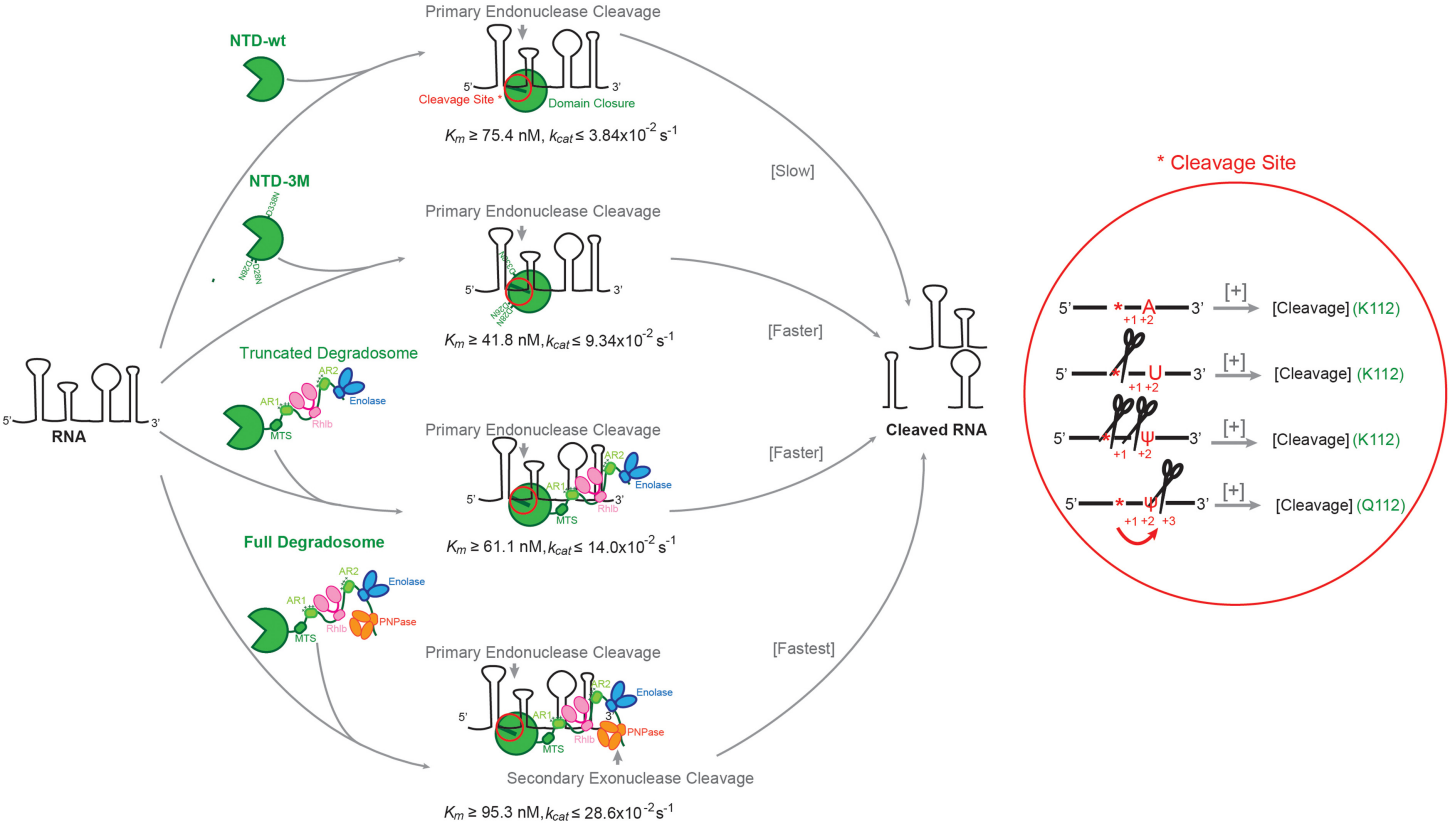
Proposed model for substrate recognition and processing by RNase E. RNase E mediated processing of RNAs within the degradosome assembly is sensitive to substrate entry and product exit where other degradosome proteins RhlB, Enolase, and PNPase play an important role. The endonuclease activity of RNase E is guided by side chain interaction with substrate and geometrical details including a hydration pattern that can be influenced by pseudouridine substitution.

Pseudouridine is a commonly occurring modification of tRNA and rRNA in all domains of life (Charette and Gray 2000). The modification of tRNA fragments with pseudouridine has been implicated in translation control in early stages of mammalian embryogenesis (Guzzi et al. 2018). In *E. coli* and other bacteria, the precursors of tRNAs and rRNAs are matured by RNase E cleavage, and the enzyme contributes to quality control of rRNA (Sulthana et al. 2016). As part of the mechanism of quality control, RNase E could hypothetically sense whether the precursors have been properly modified with pseudouridine and destroy those that have not undergone the isomerization. However, our tests of RNase E activity on tRNAs isolated from cells that are deficient in pseudouridine synthase show that these species, as well as the wild-type controls, are resistant to digestion (data not shown). Recent studies suggest that pseudouridine is also prevalent in mRNAs and noncoding RNAs, and that pseudouridylation is regulated by environmental stresses and nutrient availability (Carlile et al. 2014). Differential sensitivity of pseudouridine to ribonucleases may provide a new mechanism to control RNA stability and/or turnover. Lastly, the results presented here may offer a method to map pseudouridine positions in a sample of RNA through differential sequencing. For example, comparison of RNA sequencing of sample digested with wild-type and mutant RNase E (K112A or K112Q) might reveal attenuation of signal for substrates with uridine at position +2, but a shift of signal to the −2 or −3 position in the presence of pseudouridine (Fig. 6, right panel). This could help to pinpoint pseudouridine positions in denatured samples of cell-extracted RNA.

The degradosome scaffolding domain of RNase E is predicted to be natively unstructured, and this property has been highly sustained in evolution (Marcaida et al. 2006; Aït-Bara and Carpousis 2015). Recent findings indicate that the natively unstructured character may enable the degradosome to form microscopic condensates in the presence of RNA (Al-Husini et al. 2018, 2020), a property shared with many other RNA binding proteins from all domains of life (Lin et al. 2015; Boeynaems et al. 2018). Enzymatic activities can be concentrated within these bodies, and the environment can affect substrate RNA secondary structures (Nott et al. 2015; Guzikowski et al. 2019). The RNA degradosome from the aquatic Gram-negative bacterium *Caulobacter crescentus* coalesces into nanoscale condensates upon RNA binding, and these are reversed by RNA turnover (Al-Husini et al. 2018, 2020). Similarly, the membrane associated *E. coli* RNA degradosome forms transient clusters over the membrane during RNA turnover (Strahl et al. 2015; Moffitt et al. 2016).

The results presented here show that the catalytic power of RNase E is boosted when the enzyme is assembled into the multienzyme RNA degradosome assembly. Our observations suggest that this may arise through substrate capture by the multiple RNA binding sites in the assembly (Fig. 6, left panel). The increase in catalytic power may also be allostery-mediated. We anticipate that the clustering of degradosomes in bodies with liquid-like phase separation further concentrates the enzymatic activities of the machinery and changes the physicochemical conditions that impact on activity. Our results rationalize the origins of substrate preferences of RNase E and illuminate its catalytic mechanism, supporting the roles of allosteric domain closure and cooperation with other components of the RNA degradosome complex.

## MATERIALS AND METHODS

### RNase E NTD expression and purification

RNase E (1-529) wild-type and mutants were prepared as previously described (Callaghan et al. 2005; Bandyra et al. 2018). In brief, *Escherichia coli* strain BL21(DE3) was transformed with vector pET16 expressing RNase E (1-529) with an amino-terminal his_6_-tag. Cultures were grown in 2xTY media supplemented with 100 µg/mL carbenicillin at 37°C, in an orbital shaker set at 220 rpm. The culture was induced between 0.5 to 0.6 OD_600 nm_ by adding 1 mM isopropyl-β-thiogalactopyranoside (IPTG) and harvested after 3 h of incubation by centrifugation at 4200*g* and 4°C for 30 min. Cell pellets were stored as suspension in nickel-column buffer A (20 mM Tris pH 7.9, 500 mM NaCl, 5 mM imidazole, 1 mM MgCl_2_) at −80°C. Once thawed, the cell culture suspension was supplemented with DNase I and EDTA-free protease inhibitor cocktail tablet (Roche), and cells were lysed by passing through an EmulsiFlex-05 cell disruptor (Avestin) for 2–3 times at 10–15 kbar pressure. The lysate was clarified by centrifugation at 35,000*g* for 30 min at 4°C and the supernatant was loaded onto a preequilibrated HiTrap Chelating HP column charged with nickel ions (GE Healthcare). The column was washed extensively with wash buffer (20 mM Tris pH 7.9, 500 mM NaCl, 100 mM imidazole, 1 mM MgCl_2_), followed by linear-gradient elution of RNase E with elution buffer (20 mM Tris pH 7.9, 500 mM NaCl, 500 mM imidazole, 1 mM MgCl_2_). Fractions containing RNase E were pooled and loaded on a butyl sepharose HP column (GE Healthcare) which previously was equilibrated in high-salt buffer (50 mM Tris pH 7.5, 50 mM NaCl, 25 mM KCl, 1 M (NH)_2_SO_4_). A gradient of a low-salt buffer (50 mM Tris pH 7.5, 50 mM NaCl, 25 mM KCl, 5% glycerol) was used to elute protein. Fractions containing RNase E were pooled, concentrated and loaded onto a size-exclusion column (Superdex 200 Increase 10/300, GE Healthcare) equilibrated previously in storage buffer (20 mM HEPES pH 7.5, 500 mM NaCl, 10 mM MgCl_2_, 0.5 mM TCEP, 0.5 mM EDTA, 5% glycerol). The optimal fractions were flash frozen in liquid nitrogen and stored at −80°C until further use.

### RNase E NTD azido-phenylalanine incorporation and purification

An amber suppressor codon (TAG) was inserted by site-directed mutagenesis at defined positions of the gene encoding RNase E NTD in the pET16 expression plasmid described in the previous section. The sequences of the primers used to insert TAG codons are provided in Table 2. Para-azido-phenylalanine (p-AzidoPhe) was inserted in RNase E NTD by coexpressing in *Escherichia coli* BL21(DE3) the pET16 carrying mutated *rne* genes and pDULE2 carrying genes encoding for an orthogonal tRNA synthetase (Chatterjee et al. 2014).

**TABLE 2.**
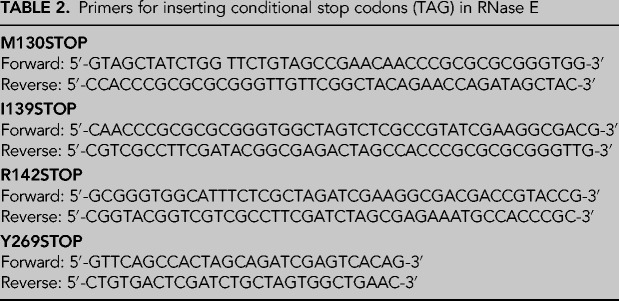
Primers for inserting conditional stop codons (TAG) in RNase E

Cultures of transformed cells were grown in LB medium supplemented with carbenicillin (100 µg/mL), spectinomycin (40 µg/mL), arabinose (0.05% w/v), and p-AzidoPhe (1 mM) at 37°C and 220 rpm. Cultures were induced between 0.5 to 0.6 OD_600nm_ by IPTG and cells were harvested by following the same procedure as used for NTD. P-AzidoPhe derivatives of NTD were purified by following the same procedure as used for NTD. The IMAC binding buffer was composed of 50 mM phosphate buffer pH 7.9, 500 mM NaCl, 5 mM imidazole, 1 mM MgCl_2_, with elution buffer containing 500 mM imidazole. The size-exclusion buffer was composed of 50 mM phosphate buffer pH 7.5, 500 mM NaCl, 10 mM MgCl_2_, 0.5 mM EDTA, 5% glycerol.

The p-azido phenylalanine incorporation was confirmed by biotinylation of azido group using EZ-link Phosphine-PEG3-Biotin (Thermo Fisher) (Saxon and Bertozzi 2000; Agard et al. 2006). Briefly, 50 µM of azido phenylalanine derivatives of RNase E NTD was reacted with 1 mM EZ-link Phosphine-PEG3-Biotin (x20 excess) at room temperature for 20 h. This allowed the phosphine group of EZ-link Phosphine-PEG3-Biotin to react with the azido group of p-azido phenylalanine, producing an aza-ylide intermediate (the Staudinger Reaction) (Saxon and Bertozzi 2000). Unbound biotin was removed by buffer exchange into phosphate buffered saline by using Micro BioSpin-6 column concentrator, followed by concentrating to 50 µL. Samples were then loaded on SDS-PAGE gel and p-azido phenylalanine was detected against anti-Biotin using a western blot transfer protocol and enhanced chemiluminescence. A similar experiment was carried out with the addition of reducing agent in the phosphate buffered saline, resulting in a less intense band. While p-azido phenyl alanine derivatives showed bands corresponding to NTD, the wild-type NTD control did not show any band with the same procedure.

### Expression and purification of truncated degradosome

*E. coli* strain ENS134-10 was used to express RNase E 1-850 and full-length RhlB genes from the expression vector pRSF-DUET and full-length enolase from pET21b. Bacterial cultures, supplemented with 15 µg/mL kanamycin and 25 µg/mL carbenicillin, were grown at 37°C until the OD_600_ reached 0.3–0.4 when protein production was induced by adding 1 mM IPTG. After overnight growth at 18°C, cells were harvested by centrifugation at 4200*g*, 4°C for 30 min. Cells were resuspended in nickel-column buffer A (50 mM Tris pH 7.5, 1 M NaCl, 100 mM KCl, 5 mM imidazole, 10 mM MgCl_2_, 0.02% n-dodecyl β-D-maltoside [β-DDM]) and stored at −80°C until further use. Once thawed, the cells were supplemented with cOmplete EDTA-free protease inhibitor tablet (Roche), 1% Triton X-100, 1 mM TCEP, 1 mM PMSF, and 100 units of DNase I. Cells were lysed by passing the suspension through an EmulsiFlex-05 cell disruptor (Avestin) for 2–3 times at 10–15 kbar pressure. The lysate was clarified by centrifugation at 35,000*g* for 30 min and the supernatant was loaded onto a preequilibrated HiTrap Chelating HP column charged with nickel ions (GE Healthcare). The column was washed extensively with wash buffer (50 mM Tris pH 7.5, 1 M NaCl, 100 mM KCl, 100 mM imidazole, 10 mM MgCl_2_, 0.02% β-DDM), followed by elution of truncated degradosome by a linear gradient of elution buffer (50 mM Tris pH 7.5, 1 M NaCl, 100 mM KCl, 500 mM imidazole, 10 mM MgCl_2_, 0.02% β-DDM). Enriched fractions evaluated by SDS-PAGE were pooled together and passed through a cation exchange column (SP HP, GE Healthcare) which previously was equilibrated in a low-salt buffer (50 mM Tris pH 7.5, 50 mM NaCl, 10 mM KCl, 0.02% β-DDM). A linear gradient (0%–50%) with a high-salt buffer (50 mM Tris pH 7.5, 2 M NaCl, 10 mM KCl, 0.02% β-DDM) was used to elute truncated degradosome. Desired fractions were pooled together, concentrated using 100 kDa molecular weight cut-off concentrator, and loaded onto a Superose6 10/300 size-exclusion column (GE Healthcare) equilibrated previously in storage buffer (50 mM HEPES pH 7.5, 400 mM NaCl, 100 mM KCl, 5 mM DTT, 0.02% β-DDM). Fractions containing the degradosome complex were flash frozen in liquid nitrogen and stored at −80°C until further use.

### Expression and purification of full degradosome

*Escherichia coli* strain NCM3416 with a chromosomally strep-tagged RNase E was used to express the endogenous full-length RNA degradosome. Bacterial cultures were grown at 37°C in 2xYT media supplemented with 50 µg/mL kanamycin until the OD_600_ reached to 2.0 when protein production was induced by adding 1 mM IPTG. After overnight growth at 18°C, cells were harvested by centrifugation at 5020*g*, 4°C for 30 min. Cells were resuspended in strep buffer A (50 mM Tris pH 7.5, 1 M NaCl, 100 mM KCl, 10 mM MgCl_2_, 0.02% β-DDM) and stored at −80°C until further use. Once thawed, the cells were supplemented with cOmplete EDTA-free protease inhibitor table (Roche), 1% Triton X-100, 1 mM TCEP, 1 mM PMSF, 100 units of DNase I, and 1 mg/mL lysozyme (Sigma). Cells were lysed by passing the suspension through an EmulsiFlex-05 cell disruptor (Avestin) for 2–3 times at 10–15 kbar pressure. The lysate was clarified by centrifugation at 35,000*g* for 30 min and the supernatant was passed through a 0.45 µ membrane filter before loading onto a preequilibrated HiTrapHP Strep column (GE Healthcare). The column was washed extensively with strep Buffer A before the endogenous RNA degradosome was step-eluted with a strep buffer B (50 mM Tris pH 7.5, 200 mM NaCl, 100 mM KCl, 10 mM MgCl_2_, 0.02% β-DDM), followed by elution of full degradosome by elution buffer (50 mM Tris pH 7.5, 1 M NaCl, 100 mM KCl, 500 mM imidazole, 10 mM MgCl_2_, 0.02% β-DDM, 2.5 mM desbiotin [Sigma]). The best fractions were pooled and applied to a cation exchange column (HiTrap Heparin HP, GE Healthcare) equilibrated in a low-salt buffer (50 mM Tris pH 7.5, 50mM NaCl, 10 mM KCl, 0.02% β-DDM). A linear gradient (0%–50%) with high-salt buffer (50 mM Tris pH 7.5, 2 M NaCl, 10 mM KCl, 0.02% β-DDM) was used to elute the full degradosome. Based on the purity of the eluted fractions, desired fractions were pooled together, concentrated down using 100 kDa MWCO concentrator, and loaded onto a Superose6 10/300 size-exclusion column (GE Healthcare) equilibrated previously in storage buffer (50 mM HEPES pH 7.5, 400mM NaCl, 100 mM KCl, 5 mM DTT, 0.02% β-DDM). Desired fractions were flash frozen in liquid nitrogen and stored at −80°C until further use.

### RNA preparation by in vitro transcription

RNAs were prepared by in vitro transcription. Plasmids with the 9S, RprA and GlmZ RNA genes were generously provided by A.J. Carpousis (CNRS, Toulouse), Kai Papenfort (Jena), and Boris Görke (Vienna), respectively. First, genes were amplified by PCR using primers which were also adding T7 promoter. Next, RNA was synthesized from the PCR amplified product using T7 RNA polymerase at 37°C, followed by treating the reaction mixture with TURBO DNase for 15–20 min at 37°C. Finally, the RNA was purified by urea-PAGE followed by electroelution at 4°C and 100V (EluTrap, Whatman) (Bandyra et al. 2018). In order to generate 5′-monophosphorylated RNA, rGMP was used in addition to rGTP (5:1 molar ratio) while keeping other reaction component and purification steps same as before (Bandyra et al. 2018). For all RNAs, purity was checked in 8% urea-PAGE gel stained with SYBRgold RNA dye (Thermo Fisher).

### RNA degradation assays with pseudouridine substrates

20-mer polyadenine (A20), polyadenine with a uracil residue at position 15 (A20U), and polyadenine with a pseudouridine residue at position 15 (A20ψ) were obtained from Dharmacon. Oligoribonucleotides were 5′ labeled with ^32^P using polynucleotide kinase (Fermentas), according to manufacturer instructions. Assays were carried out in reaction buffer (25 mM Tris-HCl pH 7.5, 50 mM NaCl, 50 mM KCl, 10 mM MgCl_2_, 1 mM DTT, 0.5 U/µL RNase OUT) at 37°C. 100 nM purified RNase E NTD was used for the reactions. Time course reactions were stopped at indicated time points by addition of STOP solution (20 mM EDTA, 2% w/v SDS). RNA loading dye (Thermo Fisher) was added to samples which were denatured (98°C, 2 min) and loaded onto polyacrylamide gels containing 7.5 M urea. Gels were dried and exposed to phosphor screens (GE Healthcare) and the signal analyzed with TyphoonT 9400 (GE Healthcare).

### Kinetics assay

Ribonuclease cleavage of RNAs by RNase E was carried out at 30°C in the reaction buffer as above (Bandyra et al. 2018). In the case of the time course assay, samples were quenched at predetermined time points by adding proteinase K mix (proteinase K in proteinase K buffer of 100 mM Tris-HCl pH 7.5, 150 mM NaCl, 12.5 mM EDTA, 1% SDS), followed by incubation at 50°C for 30 min. In the case of kinetic assay, substrate cleavage/product formation was monitored against 10, 15, 20, 25, 50, 100, 125, 150, 200, 250, 300, 350, 400, 500, 600, 700 nM of the RNA while reaction was quenched within the linear range of the time course curve (e.g., 1, 2, 3, etc. min). RNA samples were thereafter mixed with loading dye (Thermo Fisher), heated at 95°C for 2 min and loaded onto 8% urea-PAGE gel. The gels were stained by SYBR Gold (Thermo Fisher), and reaction products were visualized under a UV transilluminator (GeneSnap, Syngene). To quantify, intensity of the reaction products was calculated using GeneTools (Syngene) against a known amount of reference sample where purified 9S, p5S, and GlmZ RNAs were used as references to quantify the product/uncleaved substrate. Kinetics assay was performed for at least three time points (1, 2, 3, etc. min) and each time point is a representative of technical duplicates. Next, the reaction rate was plotted against substrate concentration using Prism (GraphPad Software).

## SUPPLEMENTAL MATERIAL

Supplemental material is available for this article.

## Supplementary Material

Supplemental Material
