# Supplementary material for: Impact of pseudouridylation, substrate fold, and degradosome organization on the endonuclease activity of RNase E
Source: RNA. 2021 Nov;27(11):1339–52. doi: 10.1261/rna.078840.121 (PMC8522691; doi:10.1261/rna.078840.121)
Supplement: Supplemental Material [file supp_27_11_1339__DC1.html]

Impact of pseudouridylation, substrate fold, and degradosome organization on the endonuclease activity of RNase E — Supplemental Material 

# Impact of pseudouridylation, substrate fold, and degradosome organization on the endonuclease activity of RNase E

## Supplemental Material

- Supplemental\_Figure\_1.pdf
