# Supplementary figures and images for: Impact of pseudouridylation, substrate fold, and degradosome organization on the endonuclease activity of RNase E
Source: RNA. 2021 Nov;27(11):1339–52. doi: 10.1261/rna.078840.121 (PMC8522691; doi:10.1261/rna.078840.121)

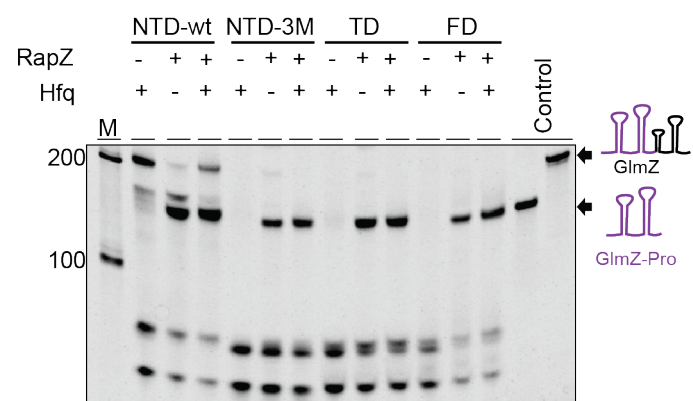

Supplement: Supplemental Material [file supp_078840.121_Supplemental_Figure_1.pdf]
